# E-MASS: Electromagnetic Mechanism for Active Shifting of the Centre of Gravity in Quadrotors Under Drive Fault

**DOI:** 10.3390/s25247679

**Published:** 2025-12-18

**Authors:** Mirosław Kondratiuk, Leszek Ambroziak, Andrzej Majka, Ranga Rao Venkatesha Prasad

**Affiliations:** 1 Department of Automation of Manufacturing Processes, Faculty of Mechanical Engineering, Bialystok University of Technology, Wiejska St. 45C, 15-351 Bialystok, Poland; m.kondratiuk@pb.edu.pl (M.K.); l.ambroziak@pb.edu.pl (L.A.); 2 Department of Aerospace Engineering, Faculty of Mechanical Engineering and Aeronautics, Rzeszow University of Technology, Powstańców Warszawy Av. 12, 35-959 Rzeszow, Poland; andrzej.majka@prz.edu.pl; 3 Networked Systems Group, Faculty of Electrical Engineering, Mathematics and Computer Science, Van Delft University of Technology, Mourik Broekmanweg 6, 2628 XE Delft, The Netherlands

**Keywords:** electromagnetic coil, permanent magnet, centre of gravity shifting, quadrotor drive system fault

## Abstract

We present a novel concept of an electromagnetic mechanism for shifting the centre of gravity (CoG) in a small unmanned aerial vehicle with four rotors (quadrotor). Shifting the CoG is essential for controlling drones in which the thrust is unbalanced (e.g., upon the failure of one of the drives). The concept presented here involves using electromagnetic coils mounted under the drone and moving permanent magnets inside a cylindrical tube. Moving the positions of the masses can be controlled by means of currents in the coils. Changing the position of the magnets relative to the arms of the drone causes a shift in the CoG, allowing for controllability even when one of the four engines is not working, and making it possible for the drone to land safely. This article describes the geometrical and mechanical relationships in the proposed system, the design and numerical calculations of the electromagnetic mechanism with coils and permanent magnets, as well as the results of a simulation of the control variant. Additionally, the practical implementation of the mechanism, from CAD modelling through the manufacturing of its elements to the final structure prepared for mounting on a quadrotor, is discussed.

## 1. Introduction

Unmanned aerial vehicles (UAVs), particularly quadrotors, are gaining popularity due to their versatility and applications in various fields, including surveillance, search and rescue, mapping, and logistics. The stability and manoeuvrability of quadrotors are crucial to the safe and effective execution of these missions, especially in conditions where normal operation is disrupted, such as in the event of partial failure of the propulsion systems.

Quadrotor UAVs are inherently susceptible to instability in the event of such failures, such as the failure of one of the rotors. Such failures often lead to significant imbalances in the thrust, causing rapid and uncontrolled rotation and potentially resulting in catastrophic failures. Diagnosing the health of the drive system and detecting potential failures is a significant engineering challenge. These issues are beyond the scope of this article, and we refer the readers to [1,2,3].

Drone control systems, among others, can focus on reconfiguring the thrust of the remaining rotors and optimising control algorithms to maintain stability [4]. These methods have limitations in cases of severe asymmetry. To enhance flight stability and maintain controllability in such adverse scenarios, a dynamic control approach for the centre of gravity (CoG) is proposed. This idea has been extensively investigated worldwide and described in numerous scientific publications [5,6,7,8,9]. By enabling dynamic adjustment of the CoG, it becomes possible to better redistribute the forces acting on the quadrotor, thereby restoring stability and increasing manoeuvrability even in emergency conditions. This approach not only aims to improve the reliability of UAV operations in failure scenarios but also lays the foundation for safer and more resilient autonomous flight systems.

This paper presents an electromagnetic system designed for real-time manipulation of the CoG, allowing the vehicle to balance asymmetric thrust conditions and maintain flight integrity. The proposed solution includes a mechanism with electromagnetic driving coils and permanent magnets that adjust the CoG of the quadrotor as they move. The proposed technique offers a reliable and easy-to-control mechanism that enhances the stability and controllability of the quadrotor.

### 1.1. Related Work

The CoG shifting problem in quadrotors manifests in several distinct forms across the literature, reflecting both deliberate design strategies and unavoidable operational disturbances. The first category involves moving mass systems, where internal mechanisms intentionally shift the CoG to support or augment attitude control. Siddhardha et al. introduced movable internal masses to achieve attitude actuation without relying solely on rotor thrust differentials, highlighting the potential of mechanical reconfiguration for robust control [10]. Similarly, Haus, Orsag, and Bogdan investigated design considerations for a large quadrotor equipped with mass-shifting modules embedded in its arms [11]. Their analysis demonstrated that moving mass control can effectively generate roll and pitch moments, yet it also introduces significant design constraints related to actuator bandwidth, power consumption, and structural integration. These works collectively illustrate that mass-shifting architectures can be intentionally leveraged as an alternative or complementary control pathway but require careful system-level trade-offs.

A second major category of CoG-related challenges arises in dynamic payload scenarios, which represent practical concerns in aerial manipulation, inspection, and cargo transport. Such scenarios include payloads that induce oscillatory or time-varying CoG shifts due to swinging, sloshing, or compliant attachments. Payload pickup and aerial manipulation introduce further complexity, as the CoG evolves during grasping, manipulation, or release tasks. In this context, Abuzayed et al. proposed a lightweight aerial manipulator with a dedicated CoG compensation mechanism designed to maintain system stability during object handling (see [8,12]). Their work emphasises that aerial manipulators inherently disrupt the nominal mass distribution during operation, requiring purposeful CoG balancing strategies to preserve controllability. Similarly, Chaisena et al. studied bounded CoG variations for known payload configurations, explicitly simulating displacements of 15, 17.5, and 20 cm to characterise the effect on stability margins and control effort [13]. These contributions underscore the importance of predictive or real-time CoG compensation in applications where the UAV interacts physically with the environment.

The third category concerns static CoG offsets, which arise not from dynamic payload changes but from structural asymmetries, design constraints, or sensor placement limitations. Such offsets persist throughout flight and may degrade control performance if unaccounted for. Kemper et al., for example, noted that practical constraints often prevent mounting sensors precisely at the true CoG, resulting in small but non-negligible offsets that propagate into state estimation and control loops [14]. These static deviations highlight the need for robust controllers capable of handling generalised CoG uncertainties without explicit mechanical compensation mechanisms.

In parallel, another stream of research examines how CoG shifts interact with faults or degradations in control effectiveness. Jia et al. investigated quadrotor safety under simultaneous CoG displacement and partial motor efficiency loss [9]. They proposed a geometric control framework augmented with a nonlinear disturbance observer capable of maintaining stable flight despite such coupled disturbances. Their findings demonstrate that CoG shifts not only affect nominal performance but can critically interact with actuator faults, motivating adaptive and fault-tolerant control strategies. Across these categories, the treatment of CoG observability varies substantially. Some approaches assume fully unknown or time-varying CoG locations, necessitating online estimation through adaptive models or coordinate-free identification laws. Others assume precisely known or simulated CoG displacements to assess controller robustness under controlled conditions. Several works also omit explicit CoG observability considerations, instead absorbing the effects into generalised disturbance terms. This diversity of assumptions reflects the broader challenge of integrating accurate physical modelling with practical sensing limitations. Overall, the literature demonstrates that CoG variations—whether intentional, incidental, dynamic, or static—constitute a critical factor in quadrotor modelling, control, and safety. Existing studies span mechanical, estimation, and control-theoretic perspectives, yet a unified framework for addressing CoG shifts in real-world UAV operations remains an open and active research topic.

### 1.2. Contributions

This work offers several original contributions to the field of fault-tolerant control and stability enhancement in multirotor aerial vehicles. The key contributions are as follows:

- A novel electromagnetic CoG shifting mechanism for quadrotors—the proposed system employs fixed coils and a permanent magnet mass sliding inside a cylindrical guide, enabling controlled CoG displacement through current modulation. To the best of our knowledge, no prior work has applied such an electromagnetic architecture to improve quadrotor safety and controllability under actuator malfunction.
- Comprehensive modelling of the geometric, mechanical, and electromagnetic behaviour—numerical calculations of the electromagnetic interactions between the coils and the permanent magnets are performed to determine feasible force ranges, achievable accelerations, and expected positional dynamics.
- Demonstration of significant CoG shifting capability and its impact on controllability—the work demonstrates that the achievable CoG displacement can be tuned through design parameters such as mass distribution, coil geometry, and current amplitude.
- Dynamic performance assessment of the CoG actuation mechanism—the study identifies key parameters affecting response time and outlines how increased current input and adjusted controller gains may improve the mechanism’s dynamics, offering guidance for future optimisation.

## 2. The Concept

**Figure 1:**
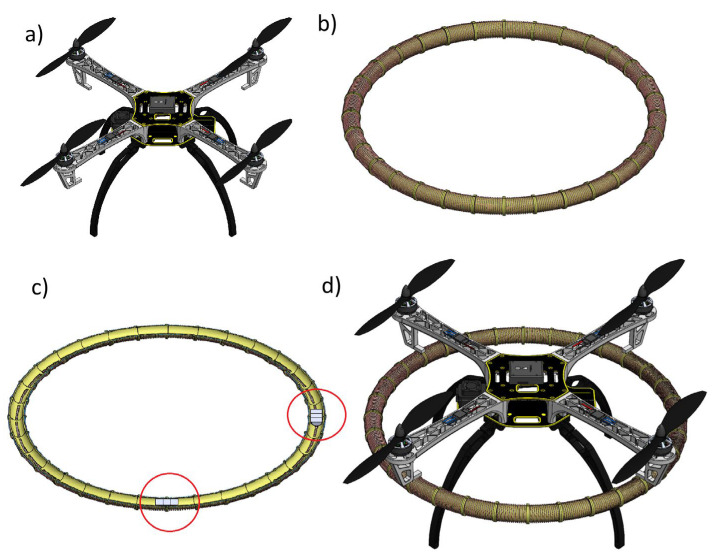
Concept of the electromagnetic mechanism for shifting the CoG and its integration with a selected drone; (**a**) CAD model of a low-cost S500 drone; (**b**) Electromagnetic coils arranged in a circle; (**c**) Horizontal cross section of the electromagnetic coils and magnets (magnets are marked in circles); (**d**) The concept of integrating a drone with E-MASS.

Figure 1a shows a CAD model of a low-cost S500 drone. Based on this model, the concept of an electromagnetic mechanism for changing CoG was developed. The idea includes mounting a mechanism with electromagnetic coils arranged in a circle under the drone’s arms (Figure 1b). Two neodymium magnets will be placed inside the coils (Figure 1c). The position of the magnets can be established by a system of optical gates placed between the coils. By switching the current in the coils, the position of the magnets can be changed and controlled. The movement of the magnets in a circle shifts the centre of gravity of the entire system. This can be used to adjust the CoG, for example, after a failure of one of the drives. The control algorithm for a drone with a shifted CoG is not part of this article, but it has been described in, among others, [9].

The proposed concept of integrating a drone with an electromagnetic mechanism for moving the CoG is shown in Figure 1d. The design of the mechanism for moving the CoG is divided into a mechanical part and an electromagnetic part.

### 2.1. Mechanical Part

**Figure 2:**
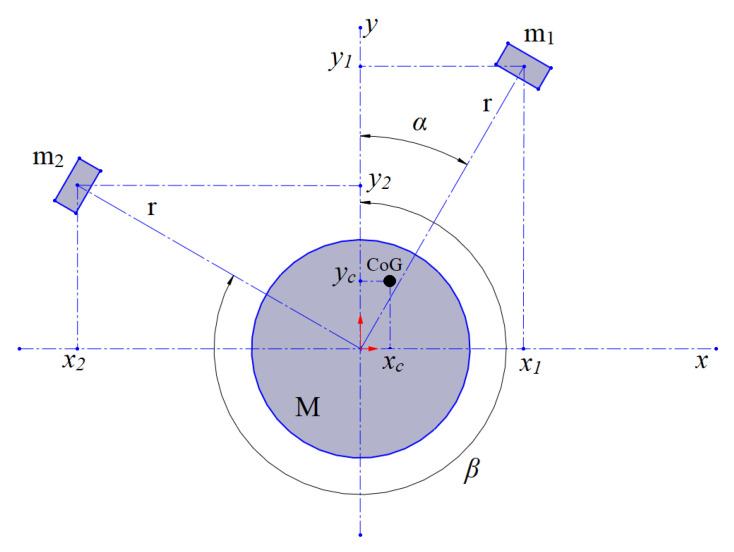
Mass distribution in the system.

In Figure 2, a diagram of the mass distribution in the system is shown. M and the masses of the magnets by *m*_1_ and *m*_2_ denote the mass of the drone and the stationary parts of the mechanism. The magnets move in a circle of radius *r*, and the angles α and β describe their position. The CoG is represented by the coordinates *x_c_* and *y_c_*.

The coordinates of the CoG can be described as follows:

(1) $$x_{c}=\frac{m_{1}rsinsin\;\left(\alpha \right)\;+\;m_{2}rsinsin\;\left(\beta \right)\;}{m_{1}\;+\;m_{2\;}+\;M},$$

(2) $$y_{c}=\frac{m_{1}rcoscos\;\left(\alpha \right)+{\;m}_{2}rcoscos\;\left(\beta \right)\;}{m_{1}+m_{2}+M}.$$

**Figure 3:**
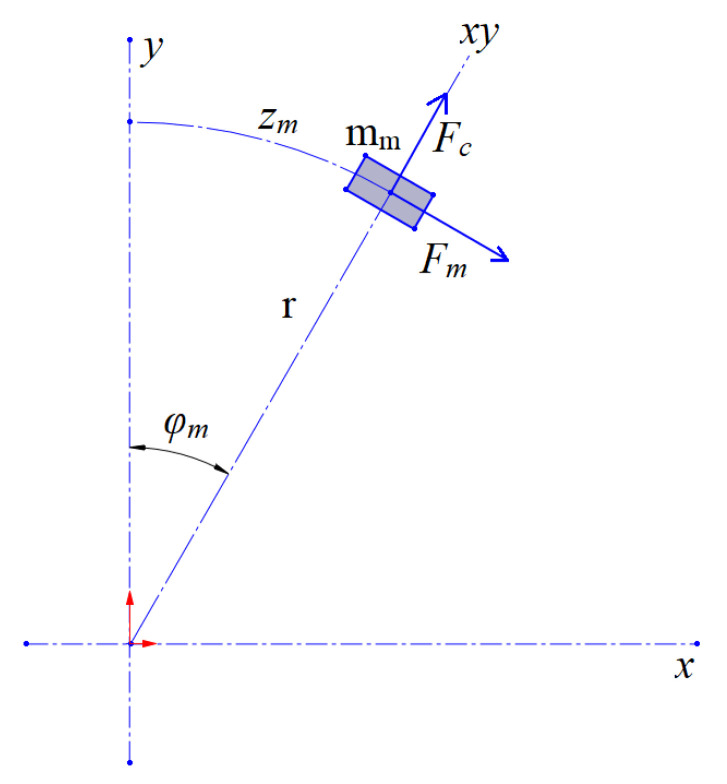
Main forces in the system.

The main forces acting on a particular magnet are presented in Figure 3.

For the sake of generalisation, the magnet’s mass is denoted by *m_m_* and the angular position of the magnet as *φ_m_*. Moreover, a linear displacement of the magnet is introduced and denoted by *z_m_*. The equation of motion for the magnet takes into account the driving force of the magnet (*F_m_*) and the repulsive force between the two magnets (*F_r_*). The magnetic force is a function of the particular coil current (*i_c_*) and the magnet’s location. Conceptually, the magnets moving in the coils are arranged in a repulsive configuration to prevent them from accidentally attracting each other. The repulsive force is a function of the linear distance between the magnets (*d_m_*). In the model, the normal force (*F_N_*), the Coulomb friction coefficient (*μ*), and the viscous damping factor (*b*) are taken into account. The equation for the dynamics of the described system is as follows:

(3) $$m_{m}{\ddot{z}}_{m}=F_{m}\left(i_{c},\;z_{m}\right)-F_{r}\left(d_{m}\right)-\mu F_{N}sign\left({\dot{z}}_{m}\right)-b{\dot{z}}_{m},$$

where *z_m_* = *rφ_m_* π/180°.

**Figure 4:**
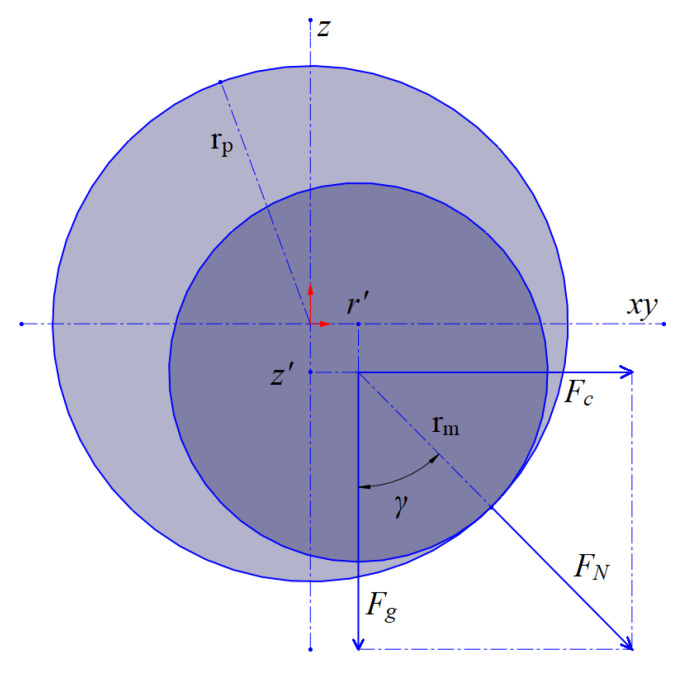
Influence of circular motion.

Cylindrical magnets of radius *r_m_* move in a circle in a tube of radius *r_p_*, as a result of which centrifugal forces appear (*F_c_*), the radius of motion increases slightly (*r*^′^), and the normal force (*F_N_*) between the magnet and the tube acts with angle (*γ*). In fact, if diameters of the tube and the magnets will be adjusted to each other (sliding motion), centrifugal forces can be simplified or even neglected. According to (3), the normal force (*F_N_*) affects the value of the Coulomb friction force. Schematically, this is presented in Figure 4.

Taking into account the acceleration due to gravity (*g*), the particular forces in the system can be written as:

(4) $$F_{g}=m_{m}g,$$

(5) $$F_{c}=\frac{m_{m}{{\dot{z}}_{m}}^{2}}{r+r^{\prime }},$$

(6) $$F_{N}=\sqrt{{F_{g}}^{2}+{F_{c}}^{2}}.$$

It is essential to note that these assumptions were made for small drone pitch and roll angles; therefore, the force due to gravity (*F_g_*) can be treated as perpendicular to the *xy* plane, and gyroscopic effects can also be neglected. In the case of significant attitude angles proposed solution cannot stabilise drone with motor malfunction, e.g., see [4]. Increasing the radius of motion of the magnets can be described by

(7) $$r^{\prime }=\left(r_{p}-r_{m}\right)sin\left(\gamma \right),$$

where *γ =* arctan(*F_c_*/*F_g_*). Increasing the radius of motion can be incorporated into a simulation of the mechanism as a correction to the coordinates of the CoG.

### 2.2. Electromagnetic Part

According to this concept, electromagnetic coils are located on a circular tube (Figure 1b), and two neodymium magnets can travel inside (Figure 1c). When we connect an electric voltage (*u_c_*) to a particular coil of resistance (*R_c_*), electric current (*i_c_*) starts to flow, and a magnetic force (*F_m_*) starts to act on the magnet in dependence on the magnet’s location (*z_m_*). Using Maxwell’s laws and based on [15,16,17], an equation for the voltage in this particular coil can be formulated:

(8) $$u_{c}=\frac{d\psi }{dt}+i_{c}R_{c},$$

where the linkage magnetic flux (*ψ*) is dependent on the current in the coil and its inductance (*L*); on the other hand, this inductance is also reliant on the coil-magnet configuration described by the magnet’s position (*z_m_*). The magnetic flux linkage to the coil-core system can be written as *ψ = i_c_L*(*z_m_*). Thus, an equation for the total voltage can be obtained:

(9) $$u_{c}=L\left(z_{m}\right)\frac{di_{c}}{dt}+i_{c}\frac{dL\left(z_{m}\right)}{dz_{m}}\frac{dz_{m}}{dt}+i_{c}R_{c}.$$

From Equation (9), it follows that in this electric system, induced voltages appear, coming from the coils’ self-inductances and from the magnet’s motion with velocity *dz_m_/dt*. This implies that the electrical power system should be appropriately designed, incorporating electrical switches (such as MOSFETs or IGBTs), safety diodes, and a current control system. This engineering problem is well-known, see, e.g., [18,19], and still widely investigated [20]; therefore, in what follows, in the case of small driving coils inductance (only several dozen coils’ turns) we assume the system is powered by a precisely controlled current (*i_c_*).

## 3. Simulation Model

To simulate this electromagnetic mechanism for shifting the CoG, we made the following design choices. Table 1 presents the crucial parameters used.

### 3.1. Numerical Analysis of the System

According to the well-known modelling approach presented, e.g., in [21,22] or the authors’ experiences, see [23], the system of coil-magnet was investigated by means of the finite element method (FEM) and COMSOL Multiphysics 6.3 software.

#### 3.1.1. Magnetic Driving Force

**Figure 5:**
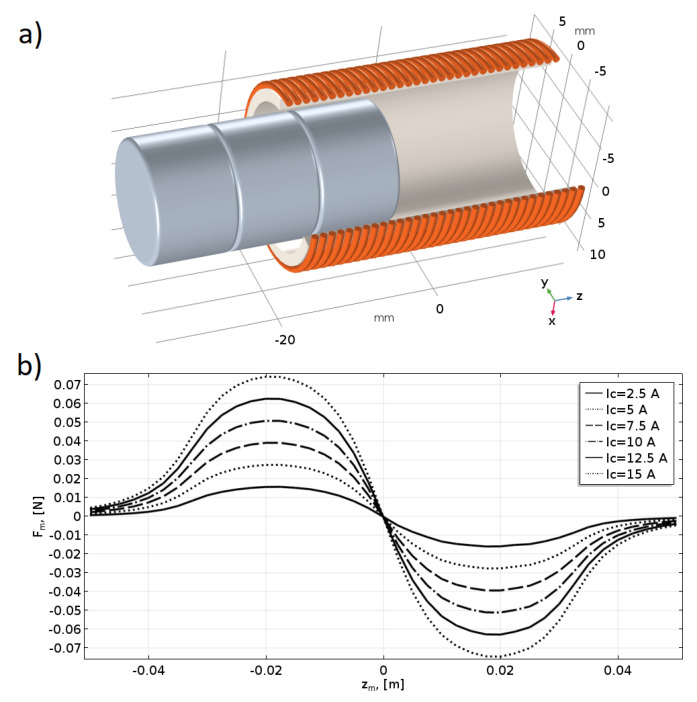
Relative forces of the coil-magnet system (using the FEM); (**a**) The geometry of the single coil with magnet; (**b**) Magnetic force values in the function of coil current and magnet position.

A model of a single coil with an axially moving magnet was developed. The conceptual curvature of the coil was neglected in the analysis due to its minimal influence on the results. In Figure 5a, the geometry of the system is visualised.

The current flow was modelled in the wires of the coil, and the Maxwell surface stress tensor (MSST) method was applied in the calculations of the magnetic force, see [15,16]. The location of the magnet, as well as the coil’s current, was parametrically changed. The results of these calculations are presented in Figure 5b. The magnetic force tries to pull the magnet to the centre of the coil. Naturally, if the direction of the current changes, the magnetic force also changes its direction. The same effect can be achieved by changing the magnet’s polarity.

The relation between the value of the magnetic force and the current in the coil can be treated as linear, see [23], so:

(10) $$F_{m}\left(i_{c},\;z_{m}\right)=i_{c}F_{m}\left(z_{m}\right).$$

This is a convenient approach because in the further stages of the analysis, the value of the current can control the magnetic force, and its relative distribution can be treated as constant for the unique coil-magnet system. In that way, the results from the FEM calculations can be directly used in dynamic simulations.

#### 3.1.2. Magnets’ Repulsive Force

**Figure 6:**
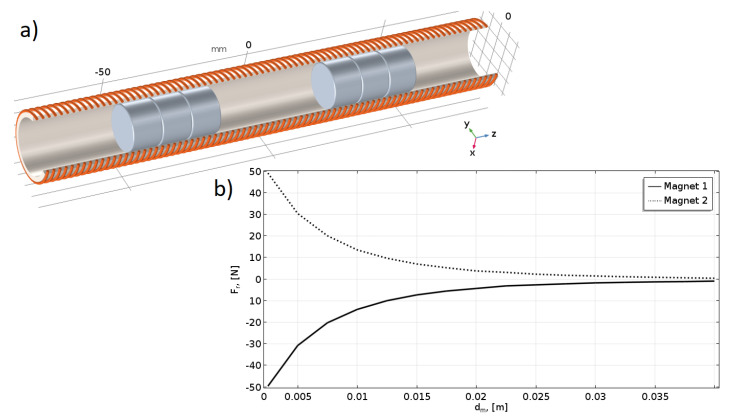
Magnets’ repelling force (using FEM); (**a**) The geometry of two axially moving magnets; (**b**) Repelling magnetic force values in the function of the distance between the magnets.

In the proposed concept, two magnets are moving in a circular tube. They should be configured in opposite polarities to avoid unintentional incidents of sticking. A model of two axially moving magnets has been developed. Figure 6a shows the geometry of the system.

To compute the repulsive force, the MSST method was applied again. The results are presented in Figure 6b. The magnets should be permanently separated, and a minimum distance between them is determined by the coils’ currents and the given characteristics of the repulsive force.

### 3.2. The Structure of the Model

**Figure 7:**
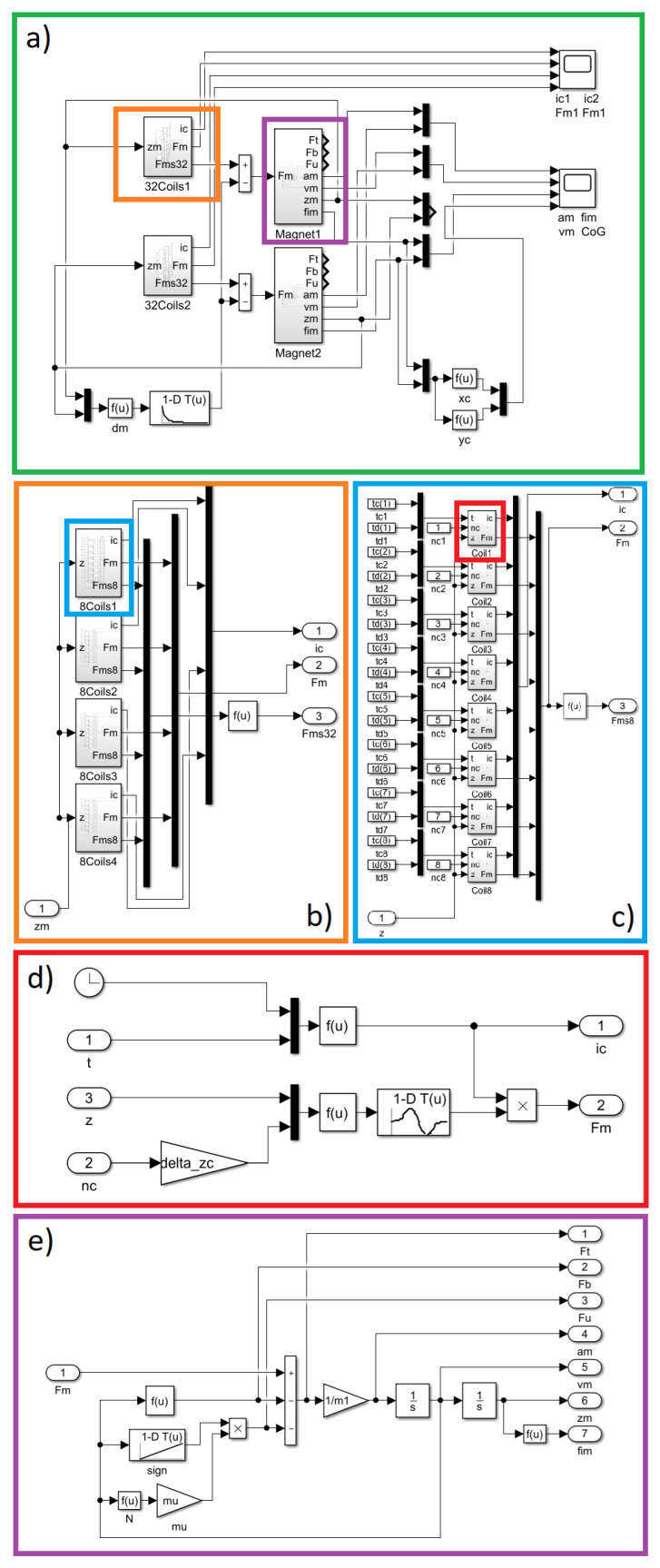
MATLAB/Simulink model implementation (**a**) A block of thirty-two coils with to magnest; (**b**) Subsystem of the 4 blocks with 8 coils; (**c**) Subsystem of single block with 8 coils; (**d**) Subsystem of magnetic force implementation; (**e**) Subsystem of magnet mechanics implementation.

To model the dynamics of the entire mechanism, MATLAB/Simulink R2024b software was utilised. The previously described equations were implemented in the simulation environment. The structures of the particular elements are presented in Figure 7. A block of thirty-two coils was introduced for each magnet to provide independent position control (Figure 7a). The configuration of blocks of the coils is presented in Figure 7b,c. The magnetic force generated in a single coil has been implemented, as shown in Figure 7d. The mechanics of a magnet were programmed, as shown in Figure 7e.

### 3.3. Initial Tests

**Figure 8:**
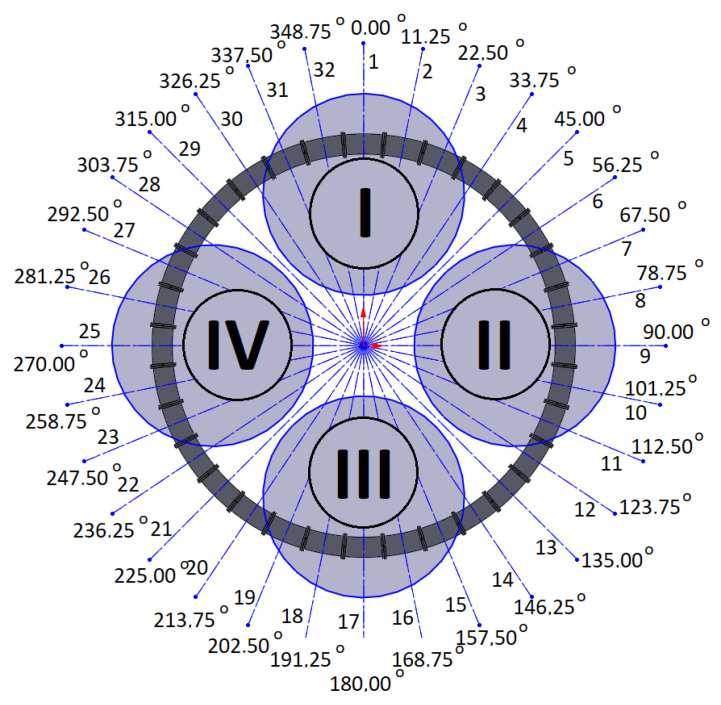
Diagram of the system with four drone motors (motors are described by Roman numerals shown in circles, black lines indicate coil separation and grey circle visualise drone rotors).

The quadrotor’s four motors (I, II, III, IV), the assigned number of coils, and their angular locations in the system are presented in Figure 8. According to the previous assumptions, the location of the CoG is described by the coordinates *x_c_* and *y_c_*, while the direction of the system’s *x*-axis coincides with 90° and the *y*-axis coincides with 0°.

The coil control algorithm requires the information of each coil’s angular location (*φ*_1_, *φ*_2_, …, *φ*_31_, *φ*_32_), the initial locations of the magnets (*φ_i_*_1_, *φ_i_*_2_), the current locations of the magnets (*φ_m_*_1_, *φ_m_*_2_), and the desired locations of the magnets (*φ_d_*_1_, *φ_d_*_2_). The current locations of the magnets can be obtained from optical gates between the coils. The concept of control is quite simple. For example, two magnets can be initially located at angular positions *φ_i_*_1_ = 90° (1st magnet) and *φ_i_*_2_ = 270° (2nd magnet). When a current is applied to coils nos 9 and 25, the magnetic force will try to hold the magnets at the centres of these coils. This is a perfect situation during a regular mission with no malfunctions of the drives in a quadrotor. When a malfunction occurs, to avoid a catastrophic accident, the quadrotor should immediately land. Shifting the CoG can make this possible safely or with minimal acceptable damage. In the case of some malfunctions of the rotor, shifting the CoG of the quadrotor can allow it to operate as a tri-rotor copter with reduced thrust and unbalanced torques (pitch, roll, and yaw). Previous work has shown that landing in such a scenario is possible [4]. To retrieve controllability, the CoG should be shifted to the desired location by putting the magnets in the appropriated positions (*φ_d_*_1_, *φ_d_*_2_); thus, a coil current control law for each particular coil can be formulated based on the current positions of each magnet (*φ_m_*), the position of the coil’s centre (*φ_c_*), and the desired position of each magnet (*φ_d_*). In the paper, the results of numerous tests were described. They were all carried out for a Coulomb friction coefficient *μ* = 0.05, damping factor *b* = 0.15 N·s/m, and for maximal coil current *i_c max_* = 10 A. Each of the tests had a slightly different control law for coil current.

#### 3.3.1. Test of the Single Coil Drive

The first test was conducted for a single-coil system with current control in the following form:

(11) $$i_{c}=\left\{\begin{array}{ccc} if\left(\varphi_{m}<\varphi_{c}\right) & \to & i_{c\;max}\\ else & \to & 0 \end{array}\right..$$

**Figure 9:**
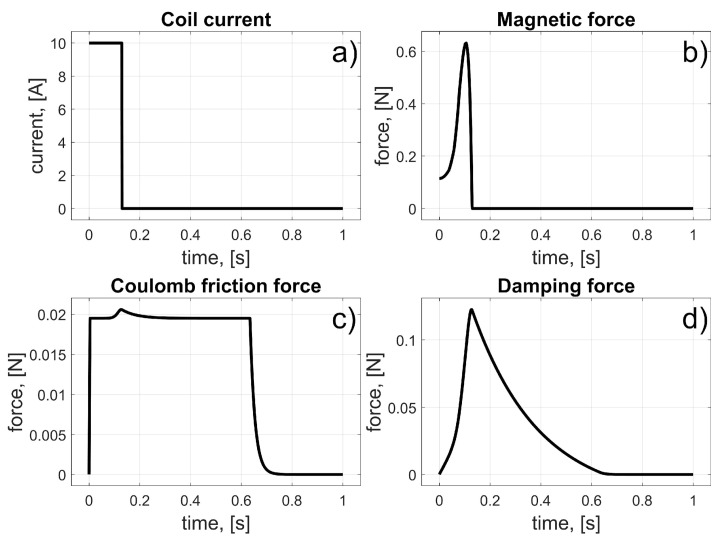
Results for the test of the single-coil drive; (**a**) Coil current; (**b**) Magnetic force; (**c**) Coulomb friction force; (**d**) Damping force.

**Figure 10:**
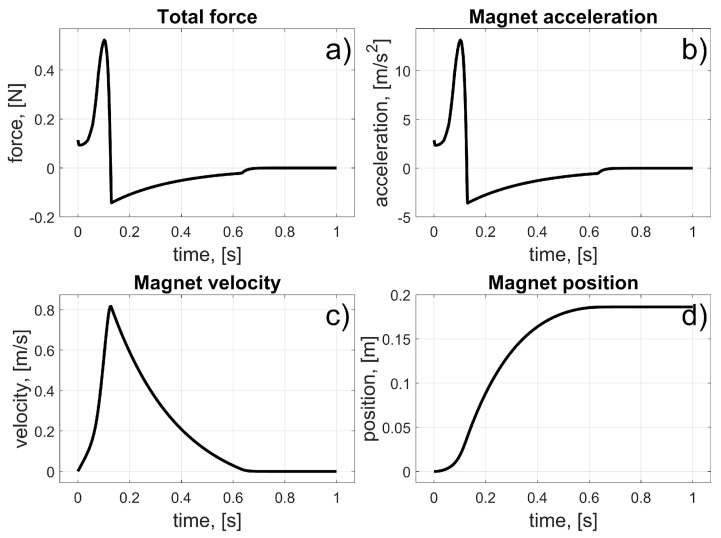
Results for the test of the single coil drive; (**a**) Total force; (**b**) Magnet acceleration; (**c**) Magnet velocity; (**d**) Magnet position.

According to (11), the coil current was turned on at the beginning and turned off when the magnet achieved a position at the centre of the coil. The initial angular position of the magnet was *φ_i_* = 0°. The coil’s angular position was *φ_i_* = 11.25° (Figure 8). All angular locations were converted into linear positions (using the radius *r*); thus, the initial linear position of the magnet was *z_i_* = 0 m and the coil’s linear position was *z_c_* = 39.27 mm. Naturally, to maintain consistency between the definition of the control law and the resultant values, the current angular position *φ_m_* of the magnet was converted to linear *z_m_*. In this way, the accelerations, velocities, and positions were presented in units of m/s^2^, m/s, and m, respectively. The results of the simulation are presented in Figure 9 and Figure 10.

From these results, we can see the influence of the frictional and damping forces. After the current starts to flow through the coil, a magnetic force is generated, and the magnet begins to move from its initial position. It reaches the centre of the coil after about 0.15 s; then, the current stops flowing, and the magnet begins to brake due to frictional forces.

#### 3.3.2. Test of the Single-Coil Braking

The second test was conducted for the single-coil system with current control in the following form:

(12) $$i_{c}=\left\{\begin{array}{c} \begin{array}{ccc} if\left(\varphi_{m}<\left(\varphi_{c}-∆\varphi \right)\right) & \to & i_{c\;max}\\ if\left(\varphi_{m}>\left(\varphi_{c}+∆\varphi \right)\right) & \to & i_{c\;max}\\ else\;\;\;\;\;\;\;\;\;\;\; & \to & 0\;\;\;\;\;\;\;\; \end{array} \end{array}\right..$$

**Figure 11:**
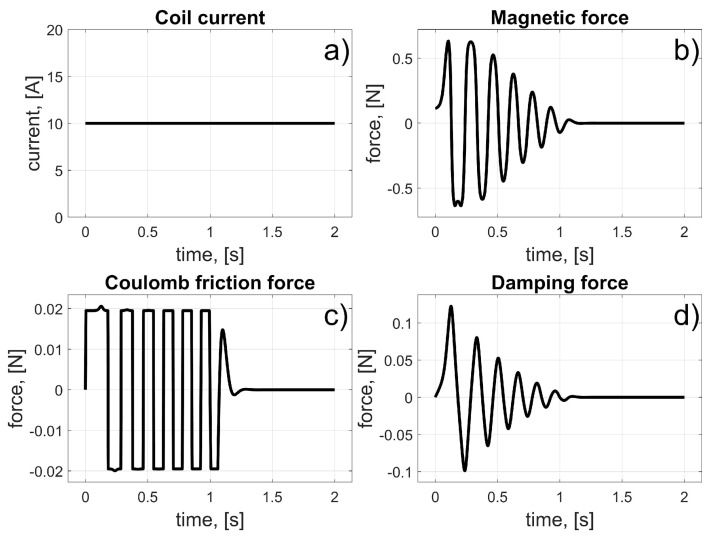
Results for single-coil braking test; (**a**) Coil current; (**b**) Magnetic force; (**c**) Coulomb friction force; (**d**) Damping force.

**Figure 12:**
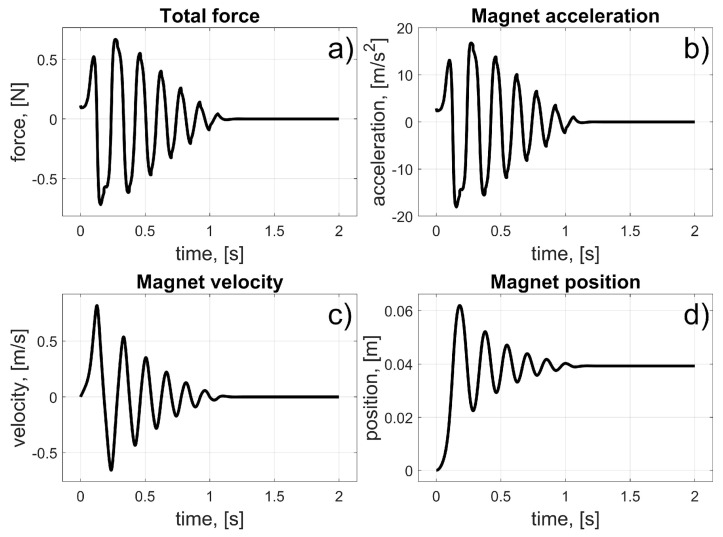
Results for single-coil braking test; (**a**) Total force; (**b**) Magnet acceleration; (**c**) Magnet velocity; (**d**) Magnet position.

According to (12), the coil current remained on when the magnet reached and crossed the centre of the coil. This causes a braking force to appear, and after some oscillations, due to the characteristics of the magnetic force, Coulomb friction, and the damping force, the magnet stops in the centre of the coil (*φ_c_*). Again, the initial linear position of the magnet was *z_i_* = 0 m, and the coil’s linear position was *z_c_* = 39.27 mm. A parameter Δ*φ* is introduced into the control law to avoid numerical instability, which, according to (3), can appear when the magnet’s velocity changes its sign. The value of Δ*φ* was arbitrarily chosen to be 1% of the coil’s angular length (11.25°, which corresponds to a linear value of 0.3927 mm). The results of the simulation are presented in Figure 11 and Figure 12.

Current flowed continuously through the coil. As the magnet began to move and reached the centre of the coil, the magnetic force began to retard its motion. Oscillations occurred, and the magnet stopped at the centre of the coil. The entire procedure took about 1 s. This test demonstrated how to stop the magnet’s motion at a desired position.

#### 3.3.3. Test of the Sequence of Coils

The third test was conducted for a sequence of coils with the current control in the following form:

(13) $$i_{c}=\left\{\begin{array}{c} \begin{array}{ccc} if\left(\left(\varphi_{m}<\varphi_{c}\right)\cap \left(\varphi_{m}<\left(\varphi_{d}-∆\varphi \right)\right)\right) & \to & i_{c\;max}\\ if\left(\left(\varphi_{m}>\varphi_{c}\right)\cap \left(\varphi_{m}>\left(\varphi_{d}+∆\varphi \right)\right)\right) & \to & i_{c\;max}\\ else & \to & 0 \end{array} \end{array}\right..$$

**Figure 13:**
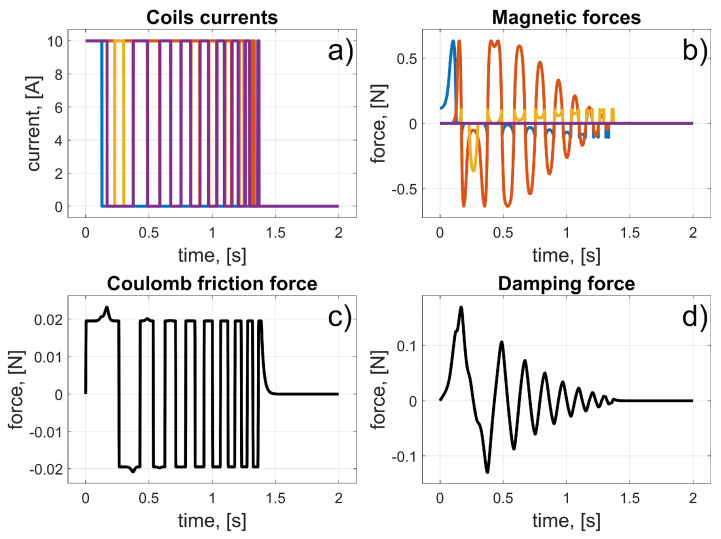
Results for coil sequence test (colours were introduced to visualize the operation of individual coils); (**a**) Coils currents; (**b**) Magnetic forces; (**c**) Coulomb friction force; (**d**) Damping force.

**Figure 14:**
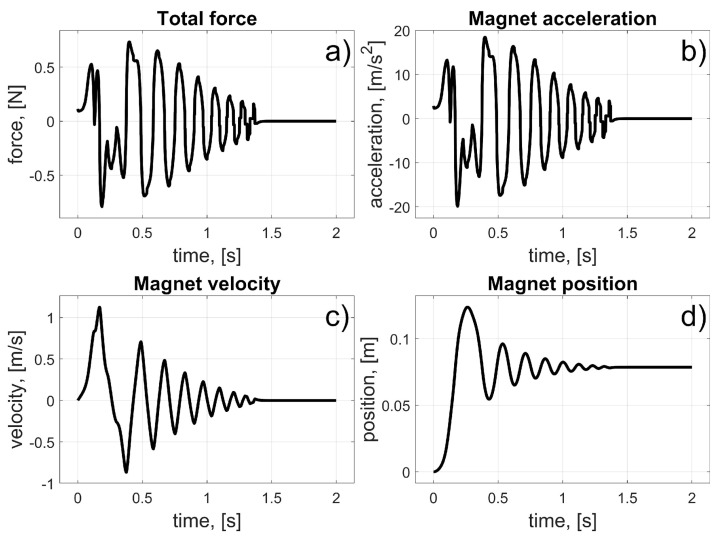
Results for coil sequence test; (**a**) Total force; (**b**) Magnet acceleration; (**c**) Magnet velocity; (**d**) Magnet position.

This examination was a kind of combination of the previous two, with coils 2, 3, 4, and 5 selected (Figure 8). The initial angular position of the magnet was *φ_i_* = 0° and the coils’ angular positions were *φ_c_*_2_ = 11.25°, *φ_c_*_3_ = 22.5°, *φ_c_*_4_ = 33.75°, and *φ_c_*_5_ = 45° (Figure 8). These correspond to *z_i_* = 0 m, *z_c_*_2_ = 39.27 mm, *z_c_*_3_ = 78.54 mm, *z_c_*_4_ = 117.81 mm, and *z_c_*_5_ = 157.08 mm. The desired angular position of the magnet was *φ_d_* = *φ*_c3_ with a linear equivalent of *z_d_* = *z_c_*_3_. The results of the simulation are presented in Figure 13 and Figure 14.

According to (13), in each coil, the current was turned on when the magnet was located before the centre. This rule was obeyed despite the direction of the magnet’s motion. If the magnet crosses the desired position, the current does not turn off, because a magnetic braking force is needed. Moreover, if the magnet crosses the desired position and, due to its inertia, draws close to the next coil, the current in that next coil is turned on after the magnet passes through the centre. In that way, the next coil supports the braking procedure. This situation is illustrated in Figure 13b.

The desired position of the magnet is the same as the position of the 3rd coil (Figure 8), so the 4th coil supports the braking process. However, the fifth coil, despite being excited by the current (purple line in Figure 13a), is too far from its desired location. Its influence is not felt (flat purple line in Figure 13b). Figure 14a,b present the total force and the magnet’s acceleration, respectively. The acceleration and braking phases are clearly visible. The entire procedure took about 1.3 s. This test demonstrated how to move a magnet to its desired position using several coils. It can be extended to thirty-two coils and two magnets.

### 3.4. Motor Malfunction Tests

The control strategy described by (13) can be applied to a complete mechanism with thirty-two coils and two magnets. The mechanism can be simulated under conditions of motor malfunctions.

The simulation scenarios involved malfunctions of specific motors. The initial positions of the magnets were set to *φ_i_*_1_ = 90° and *φ_i_*_2_ = 270°. For each different scenario of a malfunction, different desired locations of the magnets (*φ_d_*_1_, *φ_d_*_2_) were defined and the directions of shifting the CoG was obtained. It is worth mentioning that we assumed that each particular magnet moves from its initial position to its desired position (from *φ_i_*_2_ to *φ_d_*_1_ and from *φ_i_*_2_ to *φ_d_*_2_). Parameters of magnets’ desired positions for the particular simulation scenarios were presented in Table 2.

To simplify the presentation, the accelerations and velocities have been converted to their linear equivalents except for the magnets’ angular positions.

#### 3.4.1. The Malfunction of the First Motor

**Figure 15:**
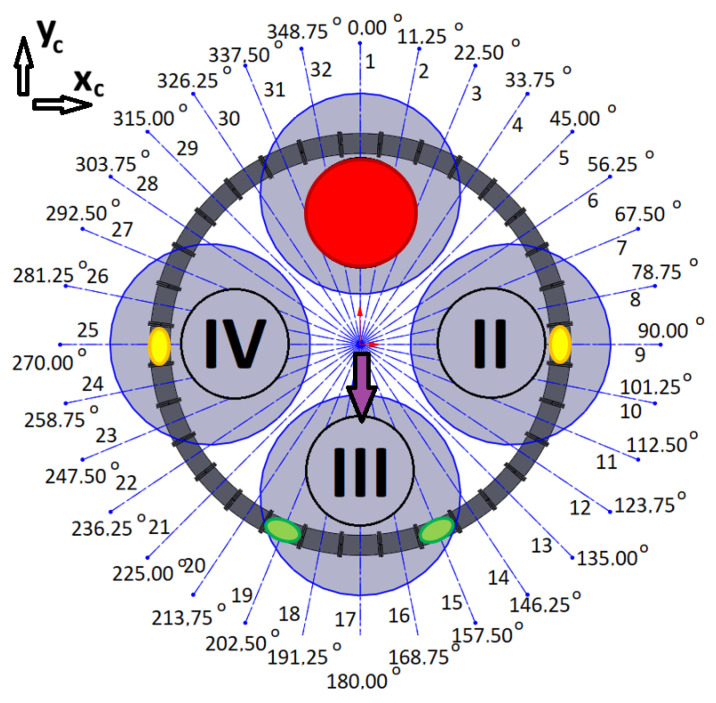
Schema for the case of a malfunction of the first motor (motors are described by Roman numerals shown in circles, black lines indicate coil separation and grey circle visualise drone rotors).

If the first motor fails (red circle in Figure 15), magnets should be shifted from initial positions (yellow spots in Figure 15) to the desired angular positions *φ_d_*_1_ = 157.5° and *φ_d_*_2_ = 202.5° (green spots in Figure 15) therefore, CoG could be shifted in the direction of 3rd motor (purple arrow in Figure 15).

**Figure 16:**
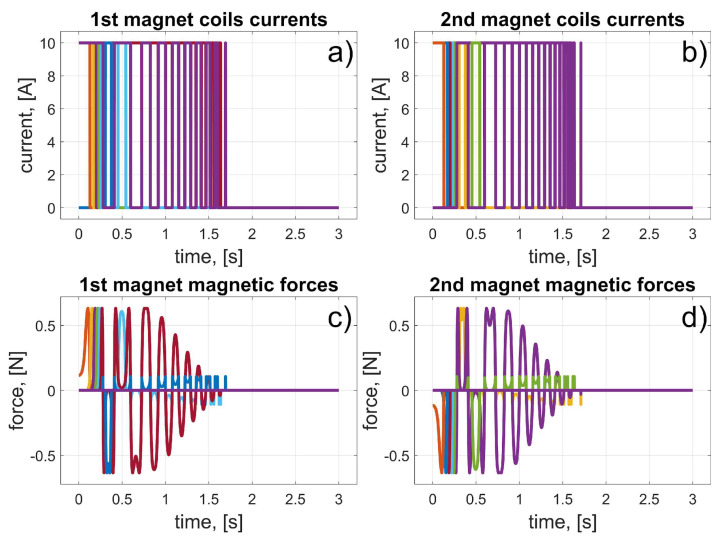
Results for a malfunction of the first motor (colours were introduced to visualize the operation of individual coils); (**a**) 1st magnet coils currents; (**b**) 2nd magnet coils currents; (**c**) 1st magnet magnetic forces; (**d**) 2nd magnet magnetic forces.

**Figure 17:**
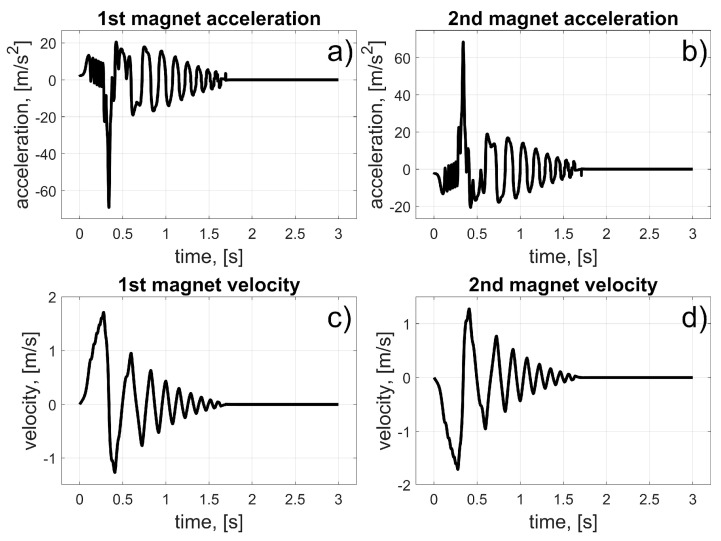
Results for a malfunction of the first motor; (**a**) 1st magnet acceleration; (**b**) 2nd magnet acceleration; (**c**) 1st magnet velocity; (**d**) 2nd magnet velocity.

**Figure 18:**
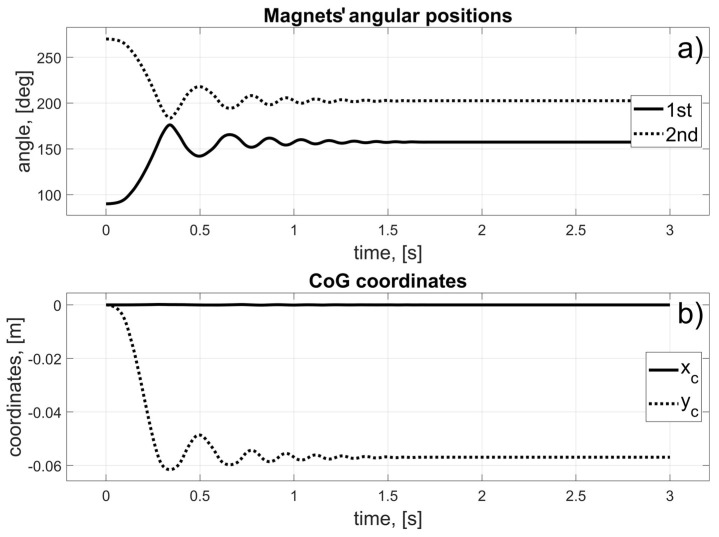
Results for a malfunction of the first motor; (**a**) Magnets’ angular positions; (**b**) CoG coordinates.

In this scenario, to move the magnets to their desired positions, coils 10 to 15 (for the first magnet), and coils 24 to 19 (for the second magnet) should be used (in fact, other coils can also be used in the braking procedure that was described in Section 3.3). The dynamic behaviour of the system is presented in Figure 16, Figure 17 and Figure 18. Because of the assumed conventions, the magnets’ motions are symmetric, and the CoG is shifted to the desired direction. The entire procedure should take about 1.5 s.

#### 3.4.2. The Malfunction of the Second Motor

**Figure 19:**
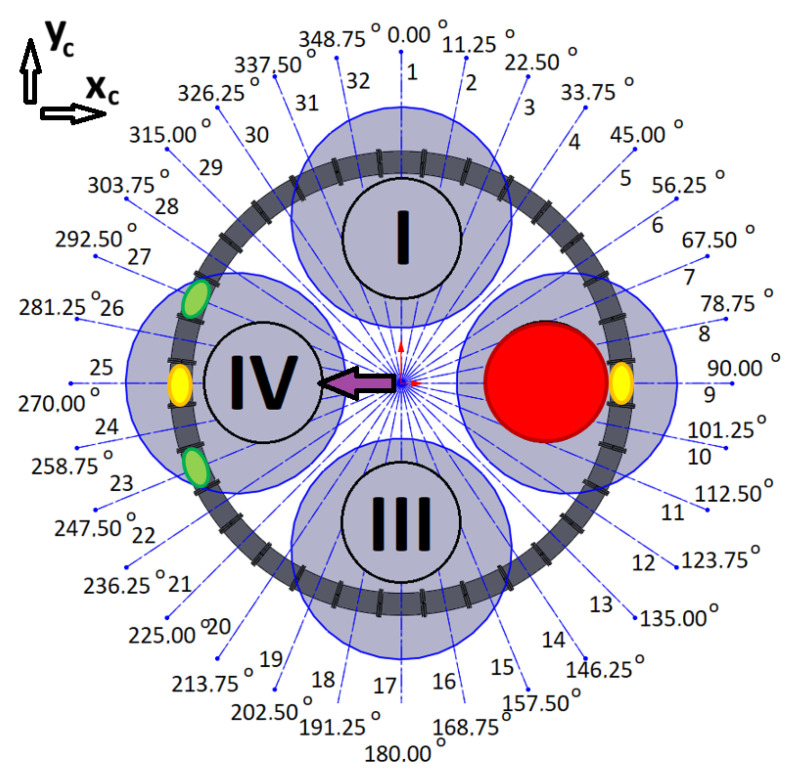
Schema for the malfunction of the second motor (motors are described by Roman numerals shown in circles, black lines indicate coil separation and grey circle visualise drone rotors).

If the second motor fails (red circle in Figure 19), magnets should be shifted from initial positions (yellow spots in Figure 19) to the desired angular positions *φ_d_*_1_ = 247.5° and *φ_d_*_2_ = 292.5° (green spots in Figure 19) therefore, CoG could be shifted in the direction of 4th motor (purple arrow in Figure 19).

**Figure 20:**
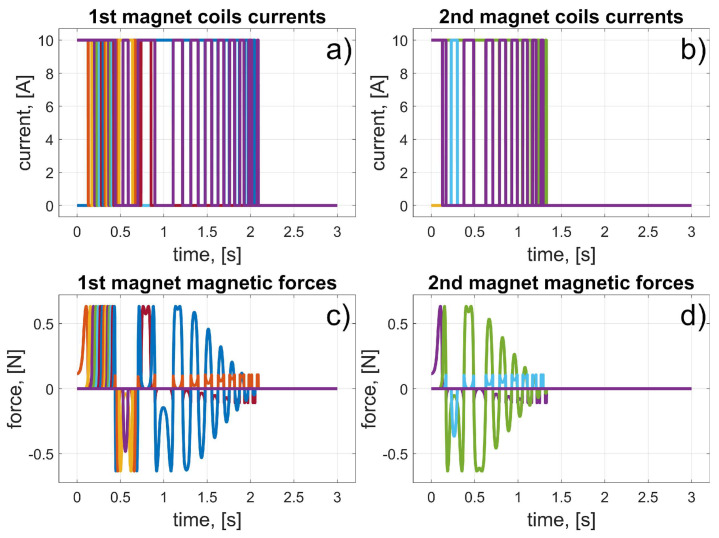
Results for malfunction of the second motor (colours were introduced to visualize the operation of individual coils); (**a**) 1st magnet coils currents; (**b**) 2nd magnet coils currents; (**c**) 1st magnet magnetic forces; (**d**) 2nd magnet magnetic forces.

**Figure 21:**
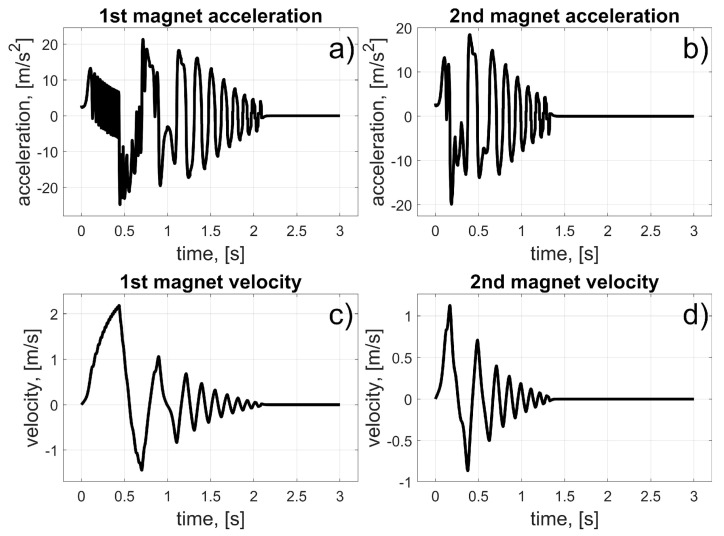
Results for malfunction of the second motor; (**a**) 1st magnet acceleration; (**b**) 2nd magnet acceleration; (**c**) 1st magnet velocity; (**d**) 2nd magnet velocity.

**Figure 22:**
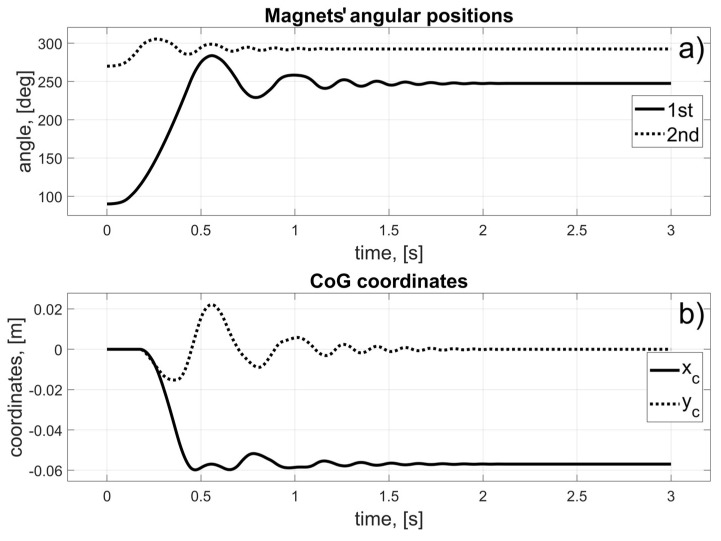
Results for the malfunction of the second motor; (**a**) Magnets’ angular positions; (**b**) CoG coordinates.

In this scenario, to move the magnets to their desired positions, coils 10 to 23 (the first magnet), and coils 26 and 27 (the second magnet) should be used. The dynamic behaviour of the system is presented in Figure 20, Figure 21 and Figure 22. In this case, the motions of the magnets are asymmetric, but the CoG is shifted to the desired direction. The entire procedure should take about 1.6 s.

#### 3.4.3. The Malfunction of the Third Motor

**Figure 23:**
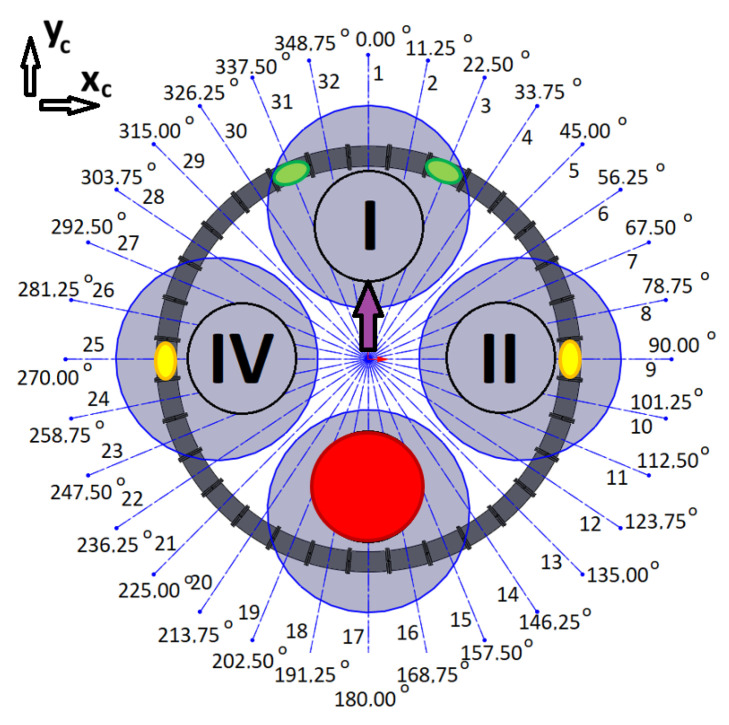
Schema for the malfunction of the third motor (motors are described by Roman numerals shown in circles, black lines indicate coil separation and grey circle visualise drone rotors).

If the third motor fails (red circle in Figure 23), magnets should be shifted from initial positions (yellow spots in Figure 23) to the desired angular positions *φ_d_*_1_ = 22.5° and *φ_d_*_2_ = 337.5° (green spots in Figure 23) therefore, CoG could be shifted in the direction of 1st motor (purple arrow in Figure 23).

**Figure 24:**
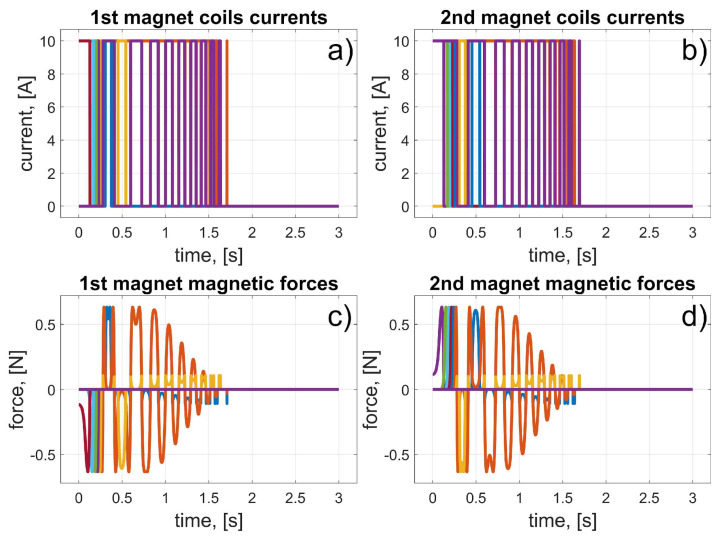
Results for malfunction of the third motor (colours were introduced to visualize the operation of individual coils); (**a**) 1st magnet coils currents; (**b**) 2nd magnet coils currents; (**c**) 1st magnet magnetic forces; (**d**) 2nd magnet magnetic forces.

**Figure 25:**
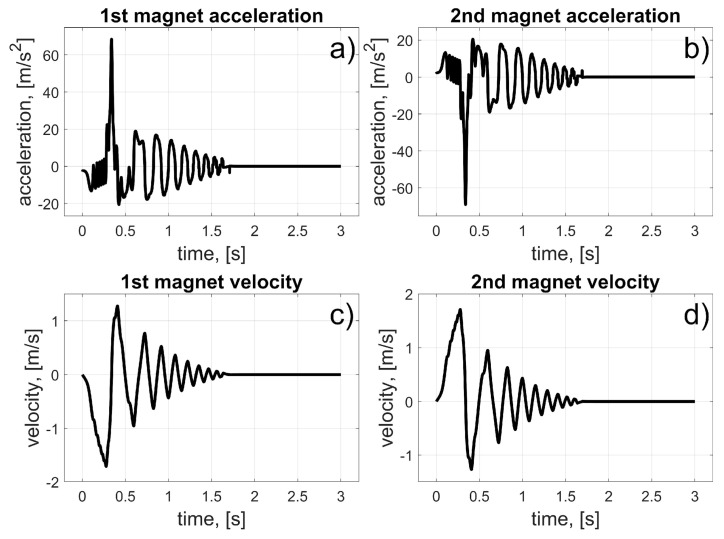
Results for malfunction of the third motor; (**a**) 1st magnet acceleration; (**b**) 2nd magnet acceleration; (**c**) 1st magnet velocity; (**d**) 2nd magnet velocity.

**Figure 26:**
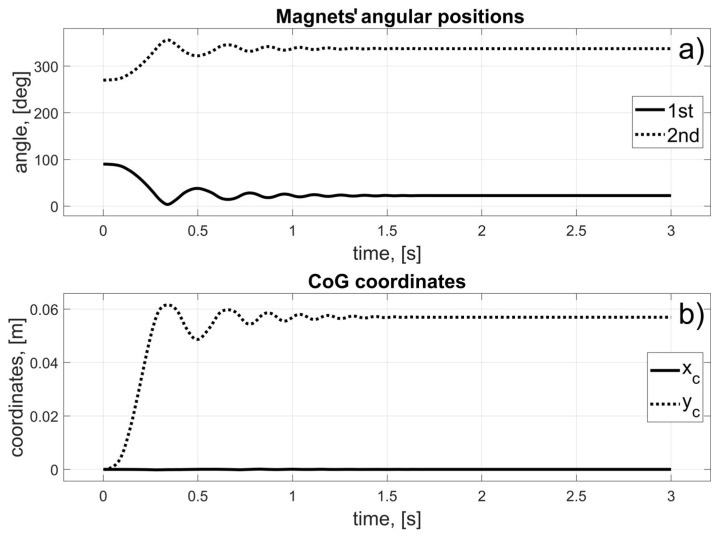
Results for the malfunction of the third motor; (**a**) Magnets’ angular positions; (**b**) CoG coordinates.

In this scenario, to move the magnets to their desired positions, coils 8 to 3 (for the first magnet) and 26 to 31 (for the second magnet) should be used. The dynamic behaviour of the system is presented in Figure 24, Figure 25 and Figure 26. The magnet’s motions are symmetric again, and CoG is shifted to the desired direction. The entire procedure should take about 1.5 s.

#### 3.4.4. The Malfunction of the Fourth Motor

**Figure 27:**
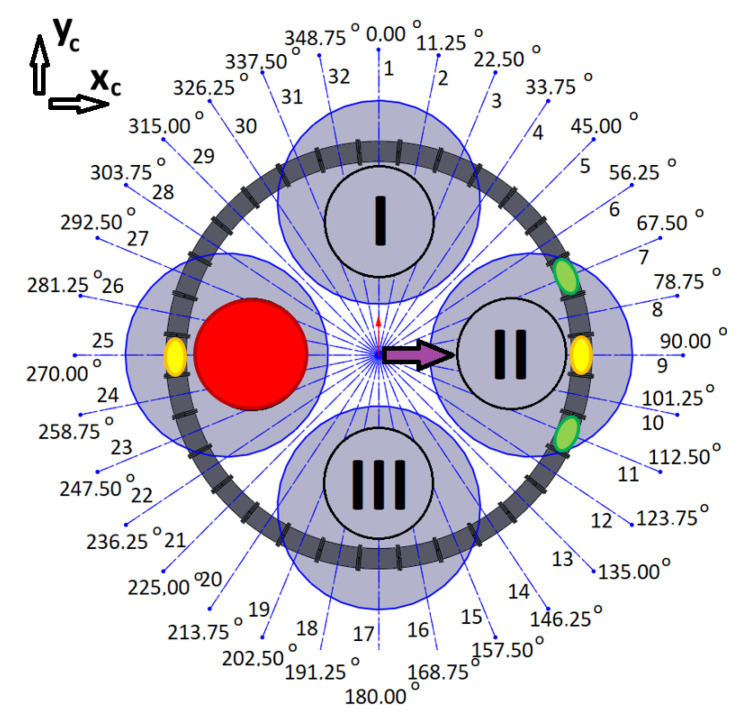
Schema for malfunction of the fourth motor (motors are described by Roman numerals shown in circles, black lines indicate coil separation and grey circle visualise drone rotors).

If the fourth motor fails (red circle in Figure 27), magnets should be shifted from initial positions (yellow spots in Figure 27) to the desired angular positions *φ_d_*_1_ = 67.5° and *φ_d_*_2_ = 112.5° (green spots in Figure 27) therefore, CoG could be shifted in the direction of 2nd motor (purple arrow in Figure 27).

**Figure 28:**
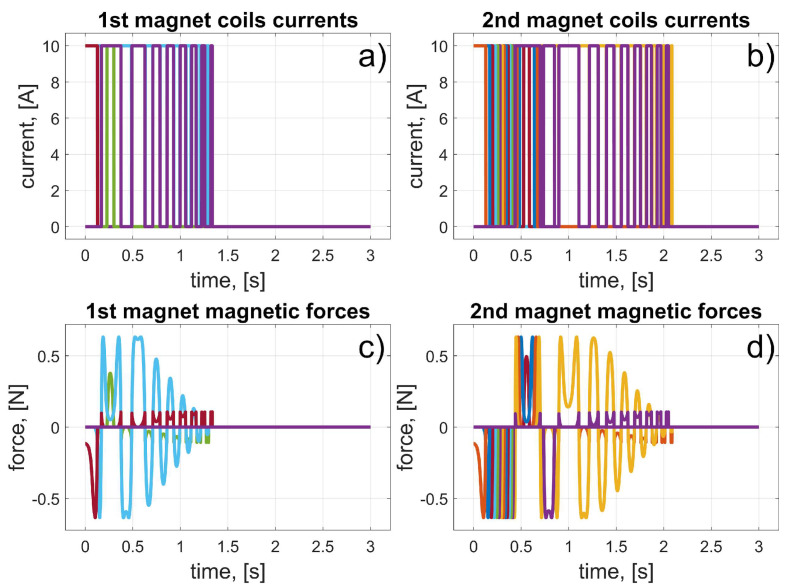
Results for a malfunction of the fourth motor (colours were introduced to visualize the operation of individual coils); (**a**) 1st magnet coils currents; (**b**) 2nd magnet coils currents; (**c**) 1st magnet magnetic forces; (**d**) 2nd magnet magnetic forces.

**Figure 29:**
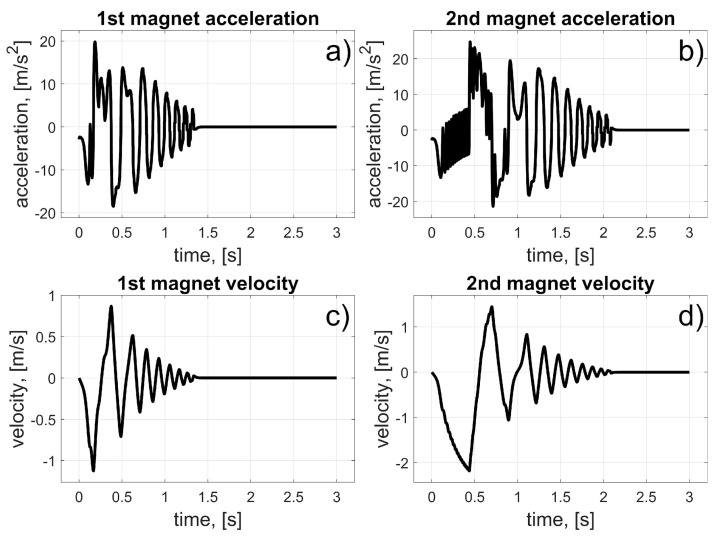
Results for a malfunction of the fourth motor; (a) 1st magnet acceleration; (**b**) 2nd magnet acceleration; (**c**) 1st magnet velocity; (**d**) 2nd magnet velocity.

**Figure 30:**
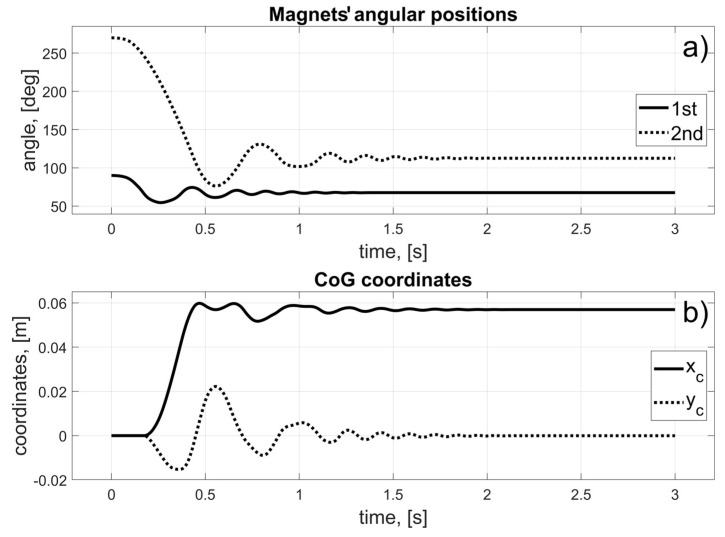
Results for a malfunction of the fourth motor; (**a**) Magnets’ angular positions; (**b**) CoG coordinates.

In this scenario, to move the magnets to their desired positions, coils 8 and 7 (for the first magnet) and 24 and 11 (for the second magnet) should be used. The dynamic behaviour of the system is presented in Figure 28, Figure 29 and Figure 30. In this case, the motions of the magnets are asymmetric, but the CoG is shifted in the desired direction. The entire procedure should take about 1.6 s.

## 4. Discussion

The simulation results obtained in this study provide a clear indication of the feasibility and practical potential of the proposed electromagnetic CoG-shifting mechanism. The presented mechanism demonstrates the ability to reposition the centre of gravity of the quadrotor by up to 6 cm, which is a substantial displacement when compared to values reported in previous research on CoG compensation for aerial vehicles. As noted in prior work, CoG shifts of this magnitude can significantly improve controllability when a rotor malfunction induces asymmetric thrust conditions. The simulations conducted herein therefore support earlier findings in the literature while showing that similar or enhanced corrective effects can be achieved using an electromagnetic actuation approach. An important aspect highlighted by the simulations is the dynamic behaviour of the mechanism, particularly the time required to reposition the magnets after a failure is detected. Across multiple malfunction scenarios, the actuation time ranged between 1.5 and 1.6 s. Although this timescale is sufficient for many emergency landing situations, it may be inadequate in cases requiring an almost instantaneous corrective response, such as small-altitude failures or aggressive flight manoeuvres. The results also identify clear engineering pathways for improving responsiveness as follows: increasing the coil current reduces actuation time, while optimising coil geometry, reducing friction, or adjusting controller gains can further enhance performance. These insights provide a roadmap for tuning the mechanism to different UAV platforms and mission profiles. Another important observation concerns the repeatability and robustness of magnet positioning under different control laws. The simulation studies involving single-coil drives, braking procedures, and multi-coil sequences demonstrate that the mechanism can reliably guide the magnets to the desired angular locations using relatively simple control strategies. This is promising for future real-time implementations, where computational constraints and the need for deterministic behaviour demand control algorithms of limited complexity. The motor malfunction scenarios further confirm that the mechanism can provide directionally appropriate CoG shifts for all possible single-rotor failures. In each of the four analysed fault cases, the magnets moved to their designated angular positions and produced the desired shift in CoG. The resulting trajectories of acceleration, velocity, and position show predictable behaviour and no signs of instability, oscillatory divergence, or failure to reach the target states. Importantly, the mechanism remained functional even under asymmetric control conditions, such as in scenarios where the magnets had to traverse differing angular distances or where braking support from neighbouring coils was necessary. From an engineering perspective, a key strength of the proposed solution lies in its lack of mechanical linkages, gears, or rotating elements, which are commonly used in mass-shifting systems and in the fact that the electromagnetic mechanism can be easily integrated into the quadrotor frame as an inherent part of its structure. The electromagnetic approach minimises mechanical wear, reduces susceptibility to backlash and transmission errors, and offers a cleaner path to miniaturisation. Moreover, because the mechanism relies primarily on electrical control, it can be integrated with existing UAV power and diagnostic systems without introducing significant structural modifications. The results also suggest broader implications for UAV safety and fault-tolerant control. Mechanisms that actively and rapidly adapt the CoG could become an important complement to existing fault-tolerant control laws, especially in severe cases where thrust redistribution alone is insufficient to maintain stability. The electromagnetic solution tested here expands the design space for such systems by providing a controllable, low-latency, and mechanically simple way to alter the mass distribution of an aircraft in emergency conditions.

## 5. Conclusions

The results of the simulations clearly show that using the proposed mechanism, the system’s CoG can be shifted into the desired direction by as much as 6 cm. According to [4,5,8,9], such a value significantly improves the controllability of a drone with a damaged drive system. Naturally, it depends on the structure of the quadrotor, as well as its mass and inertia. However, the desired parameters can be adjusted by constructional parameters.

During the simulations, the shifting procedures took about 1.5 s–1.6 s. In some cases, such a time between the detection of a rotor malfunction and the shifting of the CoG can be too long. Constructional and control parameters can be adjusted to change this. Higher currents ensure better dynamics for the motions of the magnets, see [15,16]. The proposed mechanism, to the best of our knowledge, has never been previously investigated in the field of improving the safety, stability, and controllability of a quadrotor whose drive system has malfunctioned. The results obtained strongly encourage a further examination of the proposed solution.

## 6. The Future Work

**Figure 31:**
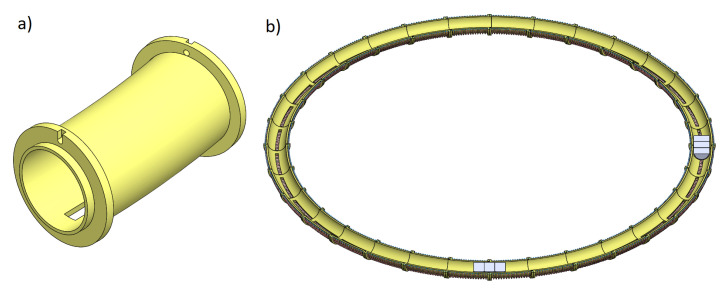
Developed CAD model of the mechanism; (**a**) Single coil base; (**b**) Horizontal cross section of the electromagnetic coils and magnets.

**Figure 32:**
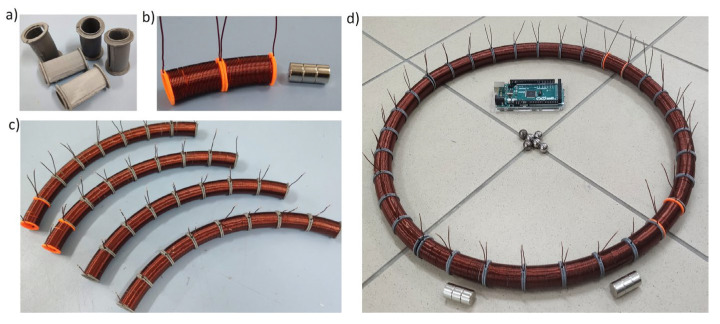
Manufacturing and assembling; (**a**) 3D printed coils’ bases; (**b**) wounded coils and magnet; (**c**) coils quarter-circle modules; (**d**) Assembled E-MASS drive system.

The simulations conducted need to be experimentally verified. Initial work on the design has already been performed. A CAD model of the system of coils was developed (Figure 31). Using 3D printing technology, the designed elements were manufactured (Figure 32a), the coils were wound (Figure 32b) and mounted as quarter-circle modules (Figure 32c). All drive systems have also been assembled (Figure 32d).

The manufactured elements are ready for the implementation of the control system. This requires some electronic components, such as optical gates, current controllers, power sources (a Li-Pol drone battery will be perfect), and a microcontroller platform for implementing the control algorithms. An experimental test of the proposed mechanism should first be conducted without quadrotors, in strictly controlled laboratory conditions. Here, some technical challenges arise, such as methods of mounting, wiring distribution, electrical noise, and interference from the potential drone-mechanism system, among others. All these should be resolved before the test stage is achieved.

**Figure 33:**
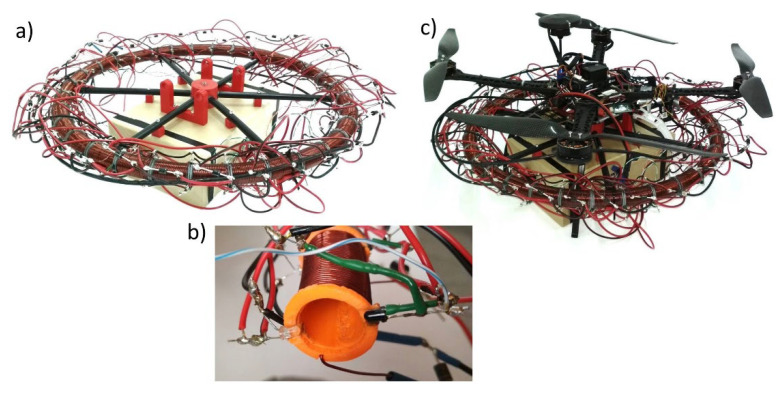
Assembled E-MASS prototype; (**a**) Assembled E-MASS drive and electronic control system; (**b**) Optical gates introduced between coils; (**c**) E-MASS prototype in the scale of S500 drone.

So far, we assembled coils and electronic control system (Figure 33a). As the magnets’ position sensors we used optical gates (Figure 33b) instead of destination continuous, e.g., Hall sensors. In Figure 33c we presented constructed mechanism in the scale of S500 drone.

**Figure 34:**
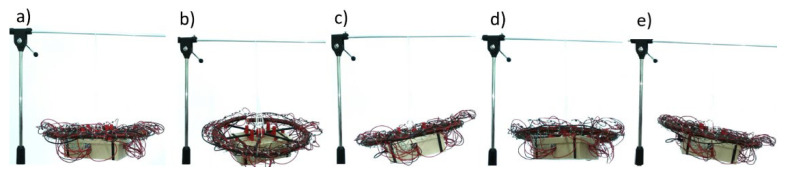
Results of initial experiments: (**a**) System in neutral position; (**b**) Scenario with 1st motor malfunction; (**c**) Scenario with 2nd motor malfunction; (**d**) Scenario with 3rd motor malfunction; (**e**) Scenario with 4th motor malfunction.

Assembled E-MASS prototype was hanged on the fixed frame. Particular coils were powered from the Li-Pol battery and coils’ currents were controlled through bipolar transistors by Arduino microcontroller with Bluetooth module. Wireless communication was implemented in order to select control algorithms during tests. Although coils’ inductances were measured only in μH, power circuits were secured by reverse diodes. Initial experiments were conducted as follows: in the microcontroller four previously described scenarios were programmed; control system was taking information of magnets’ positions and driven proper coils in sequence; each driven coil was powered for 1 s, according to the results presented in Figure 11. Thus, magnets were shifted in steps, coil by coil. For continuous motion proper sensor should be used which is connected with the nearest investigations. It is worth to mention that the total mass of the prototype was equal to 2.5 kg, including 0.5 kg of all E-MASS. In Figure 34a the prototype was presented in the neutral position.

The magnets’ motions caused mass imbalance in the system and investigated mechanism tilted to different sides depending on the chosen scenario. For scenarios of the first, the second, the third, and the fourth motor malfunction results of the initial test were presented in Figure 34b–e, respectively. Observed tilt angels indicated that designed E-MASS parameters could be enough to significantly influence to S500 drone behaviour. Future results will indicate whether the proposed mechanism achieves a field test stage with working and faulty quadrotors.

## Figures and Tables

**Table 1:** Parameters of the mechanism.

Parameter	Value
radius of mechanism (*r*)	2 cm
number of coils	32
average length of coil	39.27 mm
coil’s inner diameter	20 mm
coil’s number of turns	46
material of the coil’s wire	copper
diameter of the coil’s wire	0.85 mm
tube’s inner diameter	17 mm
magnet’s diameter	15 mm
magnet’s length	30 mm
magnet’s remanent induction	1.19 T
magnet’s mass (*m_m_*)	40 g
mass of coils and tube	0.5 kg
mass of drone (with battery pack)	2 kg
mass of non-moving parts (*M*)	2.5 kg

**Table 2:** Parameters of magnets’ desired positions for the particular simulation scenarios.

Motor Malfunction	*φ_d_*_1_ Value	*φ_d_*_2_ Value
first	157.5°	202.5°
second	247.5°	292.5°
third	22.5°	337.5°
fourth	67.5°	112.5°

## Data Availability

Data are contained within the article.
